# Gross Hematuria and Bladder Tumor in a Patient with Advanced Thyroid Papillary Carcinoma

**DOI:** 10.1155/2013/585781

**Published:** 2013-05-13

**Authors:** Takao Ando, Yuki Matsuo, Toshiyuki Ikeoka, Kojiro Oba, Yasuyoshi Miyata, Hideki Sakai, Kuniko Abe, Atsushi Kawakami

**Affiliations:** ^1^Department of Endocrinology and Metabolism, Nagasaki University Graduate School of Biomedical Sciences, 1-7-1 Sakamoto, Nagasaki 852-8501, Japan; ^2^Department of Uro-Nephrology, Nagasaki University Graduate School of Biomedical Sciences, Nagasaki 852-8501, Japan; ^3^Department of Pathology, Nagasaki University Graduate School of Biomedical Sciences, Nagasaki 852-8501, Japan

## Abstract

We present a 73-year-old female with advanced thyroid papillary carcinoma who complained of gross hematuria. We found a bladder tumor and considered it the cause of her symptom. Cystoscopic findings of the tumor were unusual, with peri-tumor vessel formation. Pathological examination of the bladder tumor was consistent with metastasis of thyroid papillary carcinoma. Therefore, we identified thyroid carcinoma metastasis to the urinary bladder as the cause of hematuria in our patient. Thyroid carcinoma metastasis to the bladder is extremely rare, but it should be included among differential diagnoses for gross hematuria in patients with a clinical history of thyroid carcinoma.

## 1. Introduction

Well-differentiated thyroid cancer is an indolent malignancy derived from the thyroid epithelial cells [1]. Common sites of distant metastasis are the lung and the bone followed by the brain [2]. Other sites of metastasis are rare and may indicate even more advanced disease. Well-differentiated thyroid cancer includes both thyroid papillary carcinoma and thyroid follicular carcinoma, the biological behaviors of which are known to differ. Thyroid follicular carcinoma tends to cause blood-borne metastasis, but thyroid papillary carcinoma tends to cause lymphatic metastasis with common recurrence in the neck lymph nodes [1]. 

## 2. Patient 

In April 2012, a 73-year-old female complained of intermittent gross hematuria. This patient had been followed in our hospital for 8 years because of her advanced thyroid cancer. Her thyroid cancer was first found in 1999, when a right lobectomy was performed. The pathological diagnosis was thyroid papillary carcinoma. She developed brain metastasis from the thyroid papillary carcinoma in 2003. The metastatic brain tumor was surgically removed, and the patient underwent whole brain irradiation. Multiple metastases in the lung were also found at the same time. The patient underwent total thyroidectomy followed by radioiodine therapy. However, there was no radioiodine accumulation other than in the thyroid bed. The patient had been maintained on TSH-suppression therapy since then; however, there had been gradual increases in serum thyroglobulin concentration as well as lung metastasis. Most recently, multiple brain metastases were identified and the patient underwent intensity-modulated radiation therapy in 2011.

**Figure 1:**
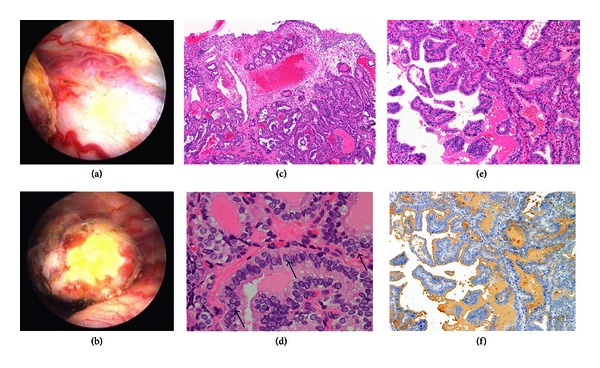
Metastatic bladder tumor of thyroid papillary carcinoma. Cystoscopy findings of the bladder tumor ((a) and (b)). It should be noted that meandering blood vessels are associated with the tumor (a). Part of the tumor was covered with a white coating (b). Pathological findings of the bladder tumor ((c) to (f)). A proliferation of atypical cells forming a papillary structure and colloid formation below the transitional epithelial cells, H&E, ×40 (c). Proliferating cells with ground-glass nuclei and nuclear groove, indicated by arrows, H&E, ×400 (d). H&E staining (e) and immunostaining with antithyroglobulin antibodies (f) of an almost identical portion of the tumor ×100.

An abdominal ultrasound study was performed in order to determine the cause of hematuria. We were able to detect a protruding mass in the bladder. CT and MRI studies showed the bladder tumor located in the right posterior wall. There was no accompanying hydronephrosis. A cytology study of the urine was negative. Since the bladder tumor was considered the cause of the gross hematuria, the patient underwent transurethral resection of the bladder tumor. Cystoscopy revealed a nonpapillary solid mass with meandering blood vessels around the tumor (Figure 1(a)). Part of the tumor was covered with a white coating which seemed to indicate necrotic change of the tumor (Figure 1(b)). Bleeding after transurethral resection seemed excessive compared to typical urothelial cancer. The postoperative course was uneventful and her hematuria disappeared.

 Pathological examination strongly suggested metastasis of the thyroid papillary carcinoma rather than primary bladder cancer (Figures 1(c) and 1(d)). The tumor was clearly positive for thyroglobulin staining (Figures 1(e) and 1(f)). Therefore, the diagnosis of thyroid papillary carcinoma metastasis to the bladder tumor was made.

## 3. Discussion

As far as we know, only two cases who developed metastatic bladder tumor caused by well-differentiated thyroid cancer have been reported [3, 4]. The patient reported by Kaplan et al. [3] had multiple pulmonary metastasis caused by thyroid follicular carcinoma. In contrast, the patient reported by Grivas et al. [4] had thyroid follicular carcinoma, but no distant metastasis other than that in the bladder. Our patient reported herein was different from those reported previously because she had advanced thyroid papillary carcinoma. 

There are several similarities in patients with metastatic bladder tumor caused by well-differentiated thyroid cancer. First, these three patients including ours were all females. This may simply reflect the female predominance of thyroid cancer [5], or that females seem to be more susceptible to metastatic bladder tumor [6]. Second, the three patients all presented with gross hematuria, similar to those with primary bladder cancer. Third, the three bladder tumors in these patients were similar in size; ~3 cm in diameter. Because blood-borne metastasis first takes place below the epithelial layer in the bladder, the metastatic tumor might have to grow to this size before it causes gross hematuria. 

The cystoscopic findings shown herein are unique and seemed to indicate hypervascularity of the metastatic tumor. Since such details were not described in the other reports [3, 4], it cannot be determined whether or not our findings are specific to metastatic thyroid cancer of the bladder. 

In conclusion, gross hematuria caused by thyroid carcinoma metastasizing to the bladder is extremely rare, but can be present as an initial symptom of distant metastasis or as one of the symptoms of advanced disease. Thyroid carcinoma metastasis to the bladder should be included among the differential diagnoses of gross hematuria, especially in female patients with a clinical history of thyroid carcinoma.
